# Genetic diversity of hepatitis C virus in Ethiopia

**DOI:** 10.1371/journal.pone.0179064

**Published:** 2017-06-01

**Authors:** Gadissa Bedada Hundie, V. Stalin Raj, Daniel GebreMichael, Suzan D. Pas, Bart L. Haagmans

**Affiliations:** 1 Department of Viroscience, Erasmus Medical Center, Rotterdam, the Netherlands; 2 National blood bank services, Ministry of Health, Addis Ababa, Ethiopia; University of Cincinnati College of Medicine, UNITED STATES

## Abstract

Hepatitis C virus (HCV) is genetically highly divergent and classified in seven major genotypes and approximately hundred subtypes. These genotypes/subtypes have different geographic distribution and response to antiviral therapy. In Ethiopia, however, little is known about their molecular epidemiology and genetic diversity. The aim of this study was to investigate the distribution and genetic diversity of HCV genotypes/subtypes in Ethiopia, using 49 HCV RNA positive samples. HCV genotypes and subtypes were determined based on the sequences of the core and the nonstructural protein 5B (NS5B) genomic regions. Phylogenetic analysis revealed that the predominant was genotype 4 (77.6%) followed by 2 (12.2%), 1 (8.2%), and 5 (2.0%). Seven subtypes were identified (1b, 1c, 2c, 4d, 4l, 4r and 4v), with 4d (34.7%), 4r (34.7%) and 2c (12.2%) as the most frequent subtypes. Consistent with the presence of these subtypes was the identification of a potential recombinant virus. One strain was typed as genotype 2c in the NS5B region sequence and genotype 4d in the core region. In conclusion, genotype 4 HCV viruses, subtypes 4d and 4r, are most prevalent in Ethiopia. This genotype is considered to be difficult to treat, thus, our finding has an important impact on the development of treatment strategies and patient management in Ethiopia.

## Introduction

Hepatitis C virus (HCV) is an important human pathogen that causes substantial morbidity and mortality worldwide. The most recent study estimated that more than 185 million people are chronically infected with HCV and 3–4 million new infections occur each year [1]. HCV is one of the leading causes of end-stage liver disease, cirrhosis and hepatocellular carcinoma [2], resulting in over 700,000 deaths annually [3]. In addition, HCV is the most common cause of death in HIV-positive patients on highly active antiretroviral therapy [4]. Consequently, the burden of HCV-related morbidity and mortality will likely continue to increase over the next twenty-five years due to the existing pool of chronic HCV infections in low-income countries [5].

HCV is an enveloped, positive-sense, single-stranded RNA virus belonging to the genus *Hepacivirus* in the family *Flaviviridae*. The viral genome is ~9.6 kb in length and contains a single open reading frame that encodes a polyprotein of about 3,000 amino acids [6]. HCV shows extreme genetic diversity and classified into seven main genotypes (1–7), further classified into 67 confirmed, 20 provisional, and 21 unassigned subtypes [7]. The HCV genotypes differ from each other by approximately 30–35%, and the subtypes differ from each other by at least 15% over complete genome [7, 8]. Different HCV genotypes and subtypes display distinct geographic distribution patterns and levels of genetic diversity [6]. Overall, genotypes 1–3 are globally distributed, causing the majority of cases in the world, whereas genotypes 4–7 are more geographically restricted [8]. Genotype 4 is found mainly in Central Africa and the Middle East [9, 10], genotype 5 in South Africa [11], and genotype 6 in Southeast Asia [12, 13]. The recently identified genotype 7 has been isolated from a Congolese immigrant in Canada [14]. This genetic diversity and geographic variation of HCV have an important impact on disease epidemiology and clinical practice because it is one of the most important predictors of response to anti-viral therapy [6, 11]. Therefore, for the development of treatment strategies and patient management, an in-depth understanding of the prevalent genotype and subtypes in different geographic regions, including Ethiopia is highly important.

Ethiopia is a large, geographically diverse nation, home to over 100 million inhabitants. Available data shows that its adult population has a low to moderate prevalence of HCV infections (0.52 to 5.8%) [15–17]. In Africa, distribution of HCV genotype exhibits two epidemiological patterns: one characterized by high genetic diversity, distributed in West Africa, with genotypes 1 and 2 [13, 18, 19], and the other in Central and Norther Africa, with genotype 4 [20]. However, little is known about the HCV genotype distribution in Eastern Africa in general and in Ethiopia in particular. The only study reporting on HCV genotypes in Ethiopia was limited to few patients attending a voluntary counseling and testing center in Addis Ababa. A total of 18 HCV RNA positive samples were analysed and a dominance of genotype 4 (50%, nine cases) followed by 2 (6 cases), 5 (2 cases) and 1 (1 case) was found [21]. However, no country-wide studies have been published and information on the HCV molecular diversity throughout the country is lacking. The aim of the present study was, therefore, to investigate the prevalence and genetic diversity of HCV genotypes and subtypes in different geographic regions of Ethiopia.

## Materials and methods

### Study area and population

This study was conducted using serum samples collected between March 2013 and April 2014 from voluntary healthy blood donors at blood bank centers in five geographic regions: Addis Ababa (the capital, central) Adama (Oromia, central-east) Gondar (Amhara, north-west) Mekelle (Tigray, north) and Jimma (Oromia, south-west). A total of 56,885 sera were collected, of which 294 were tested anti-HCV antibody positive in Ethiopia [17]. Of these, 98 sera were stored at −80°C until use and shipped to the Netherlands for further molecular study. The study was approved by the National Research Ethics Review Committee at the Federal Ministry of Science and Technology, Addis Ababa, Ethiopia. Informed written consent was given by all participants.

### Serological assays

The serological markers for HBsAg and anti-HCV antibodies were tested using commercially available enzyme-linked immunosorbent assay ELISA kits (DIALB Diagnostics GmbH, Vienna, Austria). Serum was also screened for anti-HIV-1 and -HIV-2 antibodies by ELISA (Vironostika HIV Ag/Ab, Bio-Merieux, Boxtel, The Netherlands) and anti-*Treponema Pallidum* antibodies by Rapid Plasma Reagin Test (RPR) (DIALB Diagnostics GmbH, Vienna, Austria). All tests were carried out in accordance with the manufacturers’ instructions. Anti-HCV antibody-positive serum samples from donors co-infected either with HBV or HIV were excluded from the present study. As the study subjects are healthy blood donors, clinical chemistry parameters like AST and ALT are not available.

### HCV RNA extraction, RT-PCR amplification and sequencing

HCV RNA was extracted from 140μL serum using QIAamp Viral RNA mini kit (QIAGEN, Hilden, Germany) or High Pure Viral Nucleic Acid Kit (Roche). Complementary DNA (cDNA) was synthesized using random hexamer primers (Promega, Madison, WI, USA) and SuperScriptIII (SSIII) First-Strand Synthesis System (Invitrogen) according to the manufacturers’ instruction. Briefly, the RT reaction mixture included 10μl of viral RNA, 1μl of random hexamers, 1μl of 40U/μl RNasin, 4μl of 5× First Strand Buffer, 2μl of 0.1M DTT, 1μl of dNTP mix (20 mM) and 1μl (200 U/μl) SSIII reverse transcriptase; in a final volume of 20μl. A nested PCR technique was used to amplify DNA fragments in the core and the nonstructural protein 5B (NS5B) genes. The first round of PCR was performed with 5μl cDNA in a total reaction mixture of 50μl containing 10μl of 5× HotStar HiFidelity PCR Buffer, 1μl HotStar HiFidelity DNA Polymerase (QIAGEN) and 1μl each forward and reverse outer primers (20 pmol/μl) described below. The same conditions were used in the second round PCR except 2μl of first round PCR product and 1μl each forward and reverse inner primers used here. The core gene fragment (287–751 nt, according to the position of reference HCV isolate H77 GenBank accession number AF009606) was amplified by nested PCR with outer primers s17, 410 and inner primers 953, 954, 951, as described previously [22] with some modifications. The NS5B gene (8244 to 8656 nt, according to H77) was amplified by nested PCR with a combinations of primers Pr1, Pr2, Pr3, Pr4, 122, 1204, 123 and 1203, as described previously [22, 23]. All PCR reactions were conducted with the following thermal profile 95°C for 5 min, then 40 cycles of 95°C for 1 min, 48°C/50°C for 1 min and 72°C for 1 min, with a final elongation step at 72°C for 10 min. The amplified products were gel-purified by MinElute Gel Extraction Kit (QIAGEN, Hilden, Germany) and bi-directionally sequenced using Big Dye Terminator v3.1 kit and an ABI prism 3130xl auto-sequencer (Applied Biosystems, Foster City, California, USA).

### HCV genotyping and phylogenetic analyses

Sequences were assembled and edited with SeqMan Pro (DNASTAR Lasergene 10). Comparative analyses were performed using CLUSTAL W multiple sequence alignment program, MEGA 6 software [24]. HCV genotypes and subtypes were determined phylogenetically by aligning to the reference sequences retrieved from GenBank or the Los Alamos (http://hcv.lanl.gov/content/sequence/NEWALIGN/align.html) databases and geno2pheno software (http://www.geno2pheno.org/). Bayesian inference (BI) analyses were conducted using MrBayes v3.2 [25]. For the construction of the Bayesian phylogeny, we selected the best model of nucleotide substitution based on the lowest Bayesian information criterion (BIC) and Akaike information criterion (AIC) score using TOPALi v2.5 [26]. The best model selected was a general time reversible (GTR) with a gamma-distributed rate variation across sites and a proportion of invariant sites (GTR+I+G). The Bayesian phylogenetic tree was constructed in MrBayes v3.2 with GTR+I+G model using 4 chains simultaneous run for 10 million generations, a sampling frequency of 100, and a 50% burn-in. The obtained trees were visualized in FigTree v1.4.2 [http://tree.bio.ed.ac.uk/software]. Finally, mutations in the NS5B region, codons 230–347, were assessed by geno2pheno software (http://www.geno2pheno.org/).

### Nucleotide sequence accession numbers

The nucleotide sequences of HCV core and NS5B partial regions determined in this study have been deposited in the GenBank sequence database, under the accession KY627917-KY628007.

## Results

### Demographic characteristics

A total of 98 anti-HCV positive healthy blood donors sera (51 from Addis Ababa, 13 from Adama, 15 from Gondar, 3 from Jimma and 16 from Mekelle) were analyzed in the present study. Of these, 72 (73.5%) were males and 26 (26.5%) were females. The mean age was 31. 8 years (range: 18–60 years) (Table 1).

**Table 1:** Demographic characteristics of blood donors enrolled in the study and PCR result (HCV-RNA).

Variables	Regions					Total
	Addis Ababa	Adama	Gondar	Jimma	Mekelle	
Sex						
Male	35 (68.6%)	11 (84.6%)	13 (86.7%)	2 (66.7)	11 (68.8%)	72 (73.5%)
Female	16 (31.4%)	2 (15.4)	2 (13.3%)	1 (33.3)	5 (31.2%)	26 (26.5%)
Age, years						
≤ 20	7 (13.7%)	0	2 (13.3%)	1 (33.3%)	1 (6.2%)	11 (11.2%)
21–30	14 (27.5%)	8 (61.5%)	6 (40.0%)	0	9 (56.3%)	37 (37.6%)
31–40	20 (39.2%)	3 (23.1%)	3 (20.0%)	1 (33.3%)	5 (31.3%)	32 (32.7%)
41–50	8 (15.7%)	1 (7.7%)	4 (26.7%)	1 (33.3%)	1 (6.2%)	15 (15.3%)
> 50	2 (3.9%)	1 (7.7%)	0	0	0	3 (3.1%)
PCR						
Positive	33 (64.7%)	3 (23.1%)	3 (20.0%)	2 (66.7%)	8 (50.0%)	49 (50.0%)
Negative	18 (35.3%)	10 (76.9%)	12 (80.0%)	1 (33.3%)	8 (50.0%)	49 (50.0%)

### HCV genotypes and phylogenetic analysis

HCV RNA was successfully amplified in 49 of the 98 anti-HCV-positive samples. In 49 samples, even after repeated nested PCR with a combination of different primers, no genome fragment could be amplified, which could be due to storage conditions, a high rate of false positive anti-HCV antibody results or HCV clearance. Among the PCR positive samples, 42 could be genotyped both in the NS5B and core regions, 4 in the NS5B region and 3 in the core region only. Hence, 46 and 45 samples were successfully sequenced in the NS5B and core regions, respectively (Table 2).

**Table 2:** Concordance of HCV genotypes/subtypes between core and NS5B regions.

Isolates	Genotypes/subtypes	
	Core region	NS5B region
AA328ETH	1b	1b
AA331ETH	1c	1c
AA332ETH	4d	4d
AA333ETH	4r	4r
AA345ETH	4d	4d
AA346ETH	4d	4d
AA347ETH	4d	4d
AA348ETH	4r	4r
AA350ETH	4d	4d
AA351ETH	4r	4r
AA352ETH	4d	4d
AA393ETH	4l	4l
AA394ETH	4r	4r
BL68ETH	4v	4v
BL78ETH	NA^b^	4l
JM19ETH	4d	4d
MK07ETH	4r	4r
MK08ETH	4r	4r
MK32ETH^a^	4d	2c
MK144ETH	5a	5a
AA07ETH	4d	4d
AA44ETH	4r	4r
AA58ETH	2c	2c
AA108ETH	4d	4d
AA19ETH	4r	4r
AA148ETH	4r	4r
AA55ETH	4r	4r
AA57ETH	4l	4l
AA67ETH	4d	4d
AA71ETH	NA	1b
AA214ETH	4d	4d
AA230ETH	4r	4r
AA232ETH	4d	4d
AA237ETH	4d	4d
AA174ETH	2c	2c
AA175ETH	4r	4r
AA188ETH	4r	4r
AA197ETH	4r	4r
AA239ETH	4d	4d
AA323ETH	4d	4d
GD07ETH	1b	1b
GD45ETH	2c	2c
GD93ETH	NA	2c
JM05ETH	4d	4d
BL94ETH	NA	2c
MK116ETH	4r	4r
MK58ETH	4r	NA
MK159ETH	4d	NA
MK114ETH	4d	NA

^a^Potential recombinant virus

^b^NA; not available (the genomic region couldn’t be amplified for the corresponding isolate)

**Fig 1:**
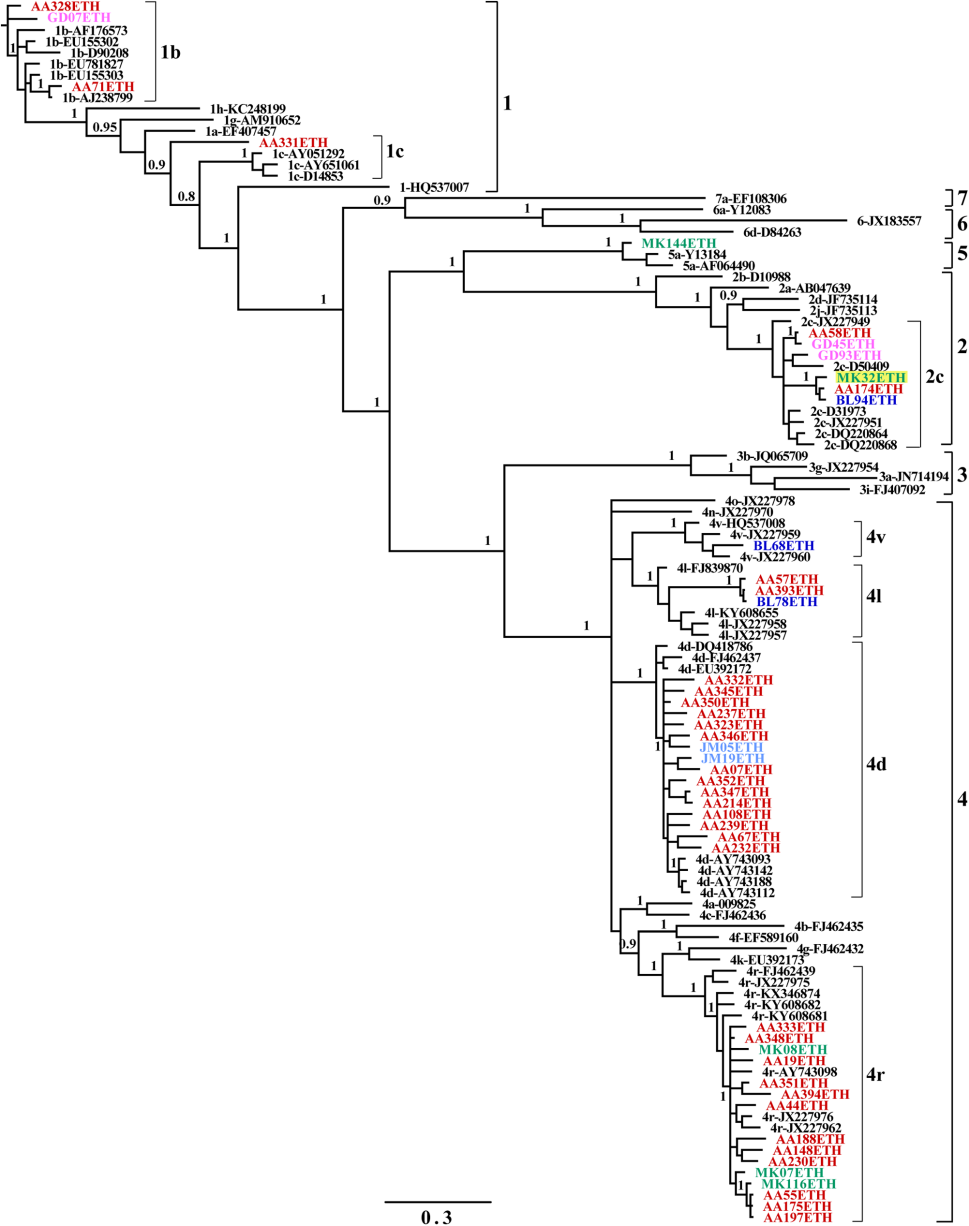
Bayesian inference phylogenetic tree of the NS5B region. A phylogenetic tree was constructed based on the NS5B region (356 nucleotides), corresponding to nucleotide numbering of 8288–8643 in the H77 genome, using the GTR+I+G model of evolution. Numbers on branches are posterior probabilities from the Bayesian inference analysis. Reference sequences are labeled to the right of each branch in the order of subtype hyphen GenBank accession number. Ethiopian sequences are shown in color (color corresponding to their geographic origin: Red = Addis Ababa; Blue = Adama; Green = Mekelle; Pink = Gondar; Light blue = Jimma), a potential recombinant MK32ETH highlighted in yellow.

**Fig 2:**
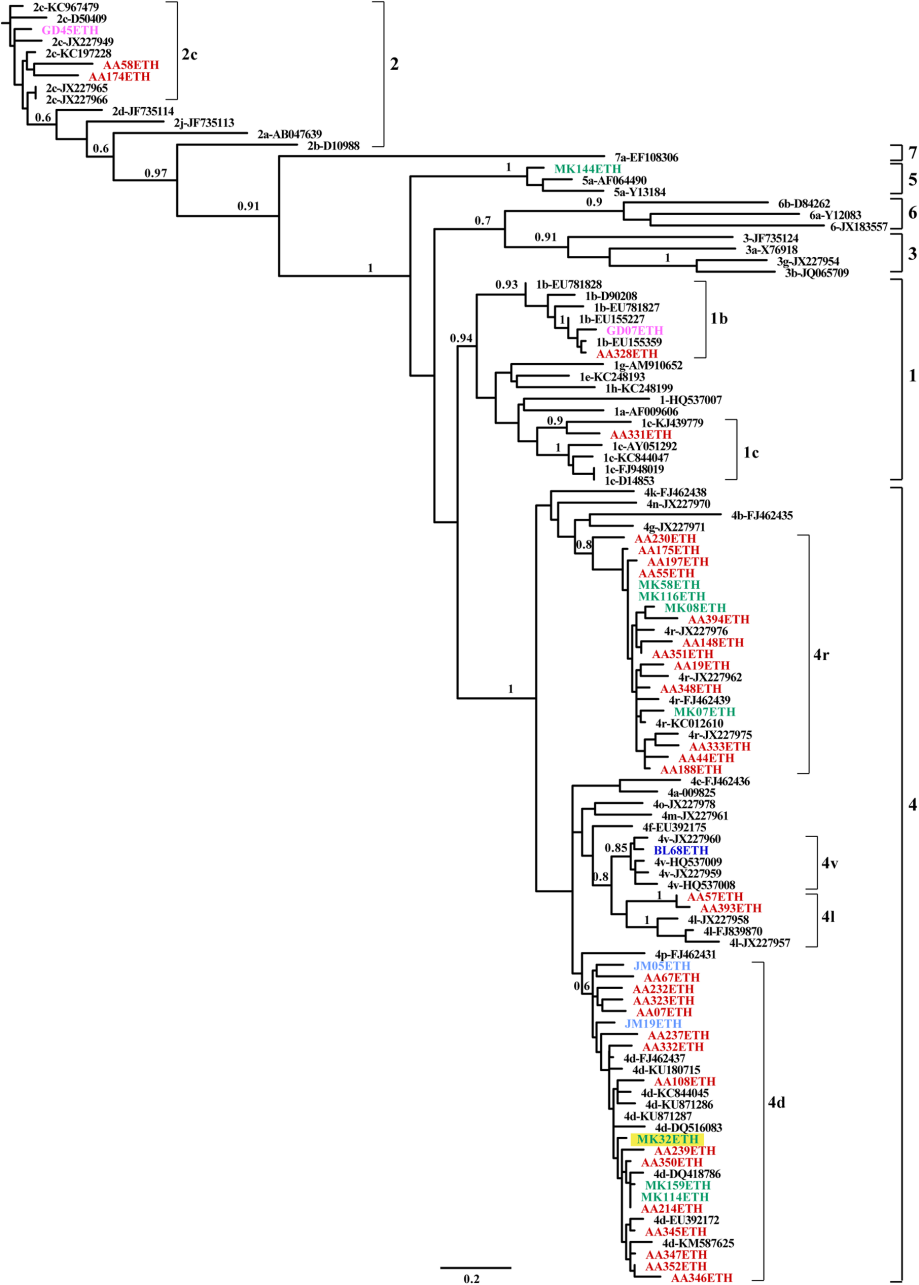
Bayesian inference phylogenetic tree of the core region. A phylogenetic tree was constructed based on the core region (418 nucleotides), corresponding to nucleotide numbering of 300–717 in the H77 genome, using the GTR+I+G model of evolution. Numbers on branches are posterior probabilities from the Bayesian inference analysis. Reference sequences are labeled to the right of each branch in the order of subtype hyphen GenBank accession number. Ethiopian sequences are shown in color (color corresponding to their geographic origin: Red = Addis Ababa; Blue = Adama; Green = Mekelle; Pink = Gondar; Light blue = Jimma), a potential recombinant MK32ETH highlighted in yellow.

A phylogenetic tree of the NS5B region was constructed of 46 Ethiopian sequences obtained in this work and reference sequences retrieved from GenBank. Four HCV genotypes (1, 2, 4 and 5) with seven subtypes (1b, 1c, 2c, 4d, 4l, 4r and 4v) were identified. As shown in Fig 1, the predominant was genotype 4, found in 35 strains (76.1%), followed by 2 (*n* = 6, 13%), 1 (*n* = 4, 8.7%), and 5 (*n* = 1, 2.2%). Of the 35 genotype 4 strains, 16 strains belong to subtype 4r, 15 strains to 4d, 3 strains to 4l and one isolate to 4v (Table 2). All genotype 2 strains belong to subtype 2c and 75% of genotype 1 strains to subtype 1b. All the genotypes and subtypes were confirmed by geno2pheno software (http://www.geno2pheno.org/). A Bayesian inference phylogenetic tree for the core region was also constructed using the 45 sequences (42 from strains used in NS5B and 3 additional strains) and reference sequences from the GenBank. The most commonly detected genotype was genotype 4 (*n* = 38, 77.6%), with predominant subtypes 4d (*n* = 18, 36.7%) and 4r (*n* = 17, 34.7%), followed by 4l (*n* = 2, 4.1%) and 4v (*n* = 1, 2.0%). Genotype 1 (*n* = 3, 6.1%) comprised the subtypes 1b (*n* = 2, 4.1%) and 1c (*n* = 1, 2.0%). Genotype 2c was represented by 3 strains (6.1%) while one strain was classified into genotype 5a (Fig 2). Genotyping was consistent when analyzed by either NS5B or core regions, except one strain (MK32ETH) which was 2c in the NS5B region but 4d in the core region, suggesting the identification of a novel recombinant HCV (Figs 1 and 2). Further full genome sequencing of this sample however failed.

Table 3 shows HCV genotype distribution in Ethiopia by age and gender. All genotype 2 strains were observed among males. Overall, no significant differences were observed between HCV genotypes with respect to donor’s age and gender.

**Table 3:** HCV genotypes distribution by age and gender № (%).

Variables	Genotype 1 № (%)	Genotype 2 № (%)	Genotype 4 № (%)	Genotype 5 № (%)	Total № (%)
**Age group**					
< 20	0	0	3 (7.9)	0	3 (6.1)
21–30	1 (25.0)	1 (16.7)	13 (34.2)	1 (100)	16 (32.7)
31–40	2 (50.0)	5 (83.3)	14 (36.8)	0	21 (42.9)
41–50	1 (25.0)	0	6 (15.8)	0	7 (14.3)
>50	0	0	2 (5.3)	0	2 (4.0)
**Gender**					
Male	2 (50.0)	6 (100)	28 (73.7)	0	36 (73.5)
Female	2 (50.0)	0	10 (26.3)	1 (100)	13 (26.5)

### Mutations in NS5B region

Mutations in the NS5B region spanning amino acid codons 230–347 were investigated using the geno2pheno software. We found that all the 46 Ethiopian sequences had mutations ranging from one to thirteen amino acid changes (S1 Table). Interestingly, the 316N variant associated with resistance to the direct acting antiviral Dasabuvir was detected in two subtype 1b strains (AA328ETH and GD07ETH) (S1 Table).

## Discussion

Knowledge of the HCV genotypes and subtypes has gained importance, because it plays a vital role in predicting the therapeutic and clinical outcome of the HCV infection [6, 27]. Moreover, the global epidemiological data show that HCV genotypes and subtypes distributions vary in different geographic regions, even among regions of the same country [11]. However, there is very limited information on the molecular epidemiology and genetic diversity of HCV infections in Eastern Africa including Ethiopia. In this study we report the first nationwide molecular epidemiology and genetic diversity of HCV in Ethiopia, one of the geographically diverse and the second most populous country in Africa. We found a high level of HCV genetic diversity, with four major genotypes (1, 2, 4 and 5) and seven subtypes circulating in the country.

Phylogenetic analysis revealed that the majority of Ethiopian HCV strains are belonging to genotype 4 (78%). This genotype is the most frequent cause of chronic hepatitis C in the Middle East, Egypt and Central Africa, accounting for more than 80% of HCV infections [28]. For instance, more than 90% of HCV infections in the Central African Republic, the Democratic Republic of Congo, Gabon and Egypt were attributed to genotype 4 [28, 29]. In Eastern Africa, however, due to the paucity of data the prevalence of HCV genotype 4 is currently not well known [20]. In recent years, the epidemiology of genotype 4 has changed and this genotype has begun spreading beyond its strongholds in Africa and the Middle East to several Western countries, particularly in Europe, due to variations in population structure, immigration and injection drug use (IDU) [9, 30]. For example, 10 to 24% of chronic HCV infections in southern Europe, particularly in France, Italy, Greece, and Spain and 11% of chronic HCV infections in The Netherlands were attributed to genotype 4 [9, 31, 32]. In addition, a high frequency of genotype 4 (42%) was found among Somali immigrants in Minnesota, USA [33]. Nowadays, a wide-ranging overview of genotype 4 epidemiology and diversity may prove useful for public health assessments not only in Africa but also outside Africa, because it accounts for 20% of total global HCV infection [20, 31, 34]. In addition, genotype 4 is considered difficult to treat and has a poor sustained virological response rate of 43–63% to the standard pegylated IFN/ribavirin combination therapy [32]. This is relatively higher than genotype 1 but lower than genotypes, 2, 3, 5, and 6 [34, 35].

Overall, the presence of four different subtypes (4d, 4r, 4l, and 4v) of genotype 4 in Ethiopia is indicative of a greater genetic diversity compared to HCV genotype 4 viruses reported in the surrounding countries. In our study, subtypes 4d and 4r are the predominant subtypes, accounting for 89% of the HCV genotype 4 infections. These subtypes are of interest because subtype 4r is not commonly reported while 4d is mostly reported in Saudi Arabia and in the majority of European IDU population [9, 28, 30, 36]. This result is thus different from studies in other African countries where subtypes 4a is the predominant subtype in Egypt [28, 35], 4f in Cameroon [37], 4e in Gabon [10, 29], 4k in the Central African Republic and Democratic Republic of the Congo [20, 38], 4c in the Republic of Congo [39], and 4q/4v in Rwanda [20]. The high diversity and predominance of genotype 4 suggests that this genotype has been endemic for a longer time in Ethiopian population. A previous study also showed the dominance of this genotype in Ethiopia [21].

The risk factors for genotype 4 and its subtypes transmission are determined by the geographical distribution of this genotype. Intravenous drug use is the most common route of transmission for genotype 4 infection in Europe while unsafe medical practice cause most cases in endemic countries [35]. In Ethiopia, although we did not assess the risk concerning genotype 4 in the present study, we suggest that sharing contaminated needles and razor blades during tattooing, body piercing, scarification, and circumcision may be the main mode of transmission, as these are common practices particularly in rural Ethiopia.

Genotype 2 was the second most prevalent HCV genotype in our study. HCV genotype 2 originated from Western Africa and disseminated to the globe through trans-Atlantic slave trade, colonial history and migrations [18, 40, 41]. In Africa, it is frequently prevalent in the west and its prevalence relative to other HCV genotypes declines from west to east [18, 41]. Our result is in agreement with the aforementioned studies. HCV genotype 2 in West Africa exhibits high genetic diversity with several subtypes identified within this genotype [18, 40]. The HCV genotype 2 from Ethiopia was found to be less diverse and belong to a single epidemic subtype 2c, suggesting that it might be introduced from Western Africa to Eastern Africa. This could be due to frequent travel of people from this geographical location to Ethiopia and vice versa.

Unlike hepatitis B virus [42], recombination between genotypes is not a common event in HCV. To date, ten HCV recombinants have been identified worldwide, of which only recombinant 2k/1b was detected multiple times and in different countries [7, 43, 44]. In the present study, we found that strain MK32ETH was characterized as genotype 2c in the NS5B gene (Fig 1) and genotype 4d in the core gene (Fig 2) as revealed by 3 different independent experiments. However, although we tried to obtain the complete genome sequence as we did previously [45], using the Bull et al. method [46], as well as 454 deep sequencing, all methods were unsuccessful.

In conclusion, this study provides important data on HCV genotypes and subtypes in Ethiopia, where four main genotypes and seven subtypes were identified. HCV infection in Ethiopia is characterized by the predominance of HCV genotype 4 (4d and 4r), with high genetic diversity, suggesting that this genotype has been endemic for a long time in Ethiopia. However, further large-scale studies on the molecular epidemiology of HCV in Ethiopia are needed. As genotype 4 is difficult to treat, our findings have a major impact in developing treatment guidelines and patient management.

## Supporting information

S1 Table Mutation in the NS5B region of HCV. (DOCX)
