# Supplementary material for: Genetic diversity of hepatitis C virus in Ethiopia
Source: PLoS One. 2017 Jun 1;12(6):e0179064. doi: 10.1371/journal.pone.0179064 (PMC5453619; doi:10.1371/journal.pone.0179064)
Supplement: S1 Table — (DOCX) [file pone.0179064.s001.docx]

| Ethiopian Sequence | subtype | Mutation in NS5B region | Dasabuvir resistance-associated mutation | Sofosbuvir resistance-associated mutation |
| --- | --- | --- | --- | --- |
| AA328ETH | 1b | C316N | 316N | none |
| AA331ETH | 1c | A238T, K254R, V262I, E272Q, L293I, T300A, S335A, A338G | none | none |
| AA332ETH | 4d | F285Y, S300T, I303T, D327N | NA* | none |
| AA333ETH | 4r | T254S, K270R, C316H, V321I | NA | none |
| AA345ETH | 4d | S254A, F285Y, S300N | NA | none |
| AA346ETH | 4d | S300T | NA | none |
| AA347ETH | 4d | A255S, S300NT, I303IT, D310DN, G328S | NA | none |
| AA348ETH | 4r | A252AV, C316H, V321I, E327AV | NA | none |
| AA350ETH | 4d | V252AV, S300T, I303IV, S347P | NA | none |
| AA351ETH | 4r | K270R, C316H, V321I, E327AE | NA | none |
| AA352ETH | 4d | S300T, I303T, D327E | NA | none |
| AA393ETH | 4l | E237G, E248D, A249T, K270R, F285L, M300T, G327D, G333T | NA | none |
| AA394ETH | 4r | K270R, C316H, V321I, E327A, A333E | NA | none |
| BL68ETH | 4v | D244N, K251R, T254D, V284T | NA | none |
| BL78ETH | 4l | E237G, E248D, A249T, K270R, F285L, M300T, G327D, G333T | NA | none |
| JM19ETH | 4d | S300T, I303T, D327G | NA | none |
| MK07ETH | 4r | C316H, V321I, E327A | NA | none |
| MK08ETH | 4r | C316H, V321I | NA | none |
| MK32ETH | 2c | T235N, N291S, K300R, V329A, R337K | NA | none |
| MK144ETH | 5a | A251V, K307R | NA | none |
| AA07ETH | 4d | D244N, I276T, S300T, I303T, D327G | NA | none |
| AA44ETH | 4r | C316H, V321I, E327A | NA | none |
| AA58ETH | 2c | K300R, V329A | NA | none |
| AA108ETH | 4d | S300T, I303V, R304K, A335S, Y346N | NA | none |
| AA19ETH | 4r | A252V, T303I, K309R, C316H, V321I, E327A | NA | none |
| AA148ETH | 4r | K270R, C316H, V321I, E327A, Y346N | NA | none |
| AA55ETH | 4r | C316H, V321I, E327A, Y346N | NA | none |
| AA57ETH | 4l | E237G, E248D, A249T, K270R, F285L, M300T, G327D, G333T, Y346N | NA | none |
| AA67ETH | 4d | K270R, I276T, F285Y, S300T, Y346N | NA | none |
| AA71ETH | 1b | S300A, D318H, D319H, A333E, R337W, V338A, Y346N | none | none |
| AA214ETH | 4d | S300T, I303T, G328S, Y346N | NA | none |
| AA230ETH | 4r | C316H, V321I, E327A, Y346N | NA | none |
| AA232ETH | 4d | I276T, S300T, Y346N | NA | none |
| AA237ETH | 4d | S300T, D310N, V322I, Y346N | NA | none |
| AA174ETH | 2c | T235N, T267R, K300R, V329A, R337K, Y346N | NA | none |
| AA175ETH | 4r | C316H, V321I, E327A, Y346N | NA | none |
| AA188ETH | 4r | 247T, K270KR, C316H, V321I, E327A, D332N, R334E, A335P, T340P, R345G, Y346N | NA | none |
| AA197ETH | 4r | C316H, V321I, E327A, Y346N | NA | none |
| AA239ETH | 4d | K270R, I276T, S300T, I303T, Y346N | NA | none |
| AA323ETH | 4d | S300T, I303T, D327G, M343I, Y346N | NA | none |
| GD07ETH | 1b | A252V, R254K, Q309R, C316N | 316N | none |
| GD45ETH | 2c | A299D, K300R, V309IV, V329A | NA | none |
| GD93ETH | 2c | L241Q | NA | none |
| JM05ETH | 4d | S269G, T286P, L293M, S300T | NA | none |
| BL94ETH | 2c | T235N, T267R, K300R, R337K | NA | none |
| MK116ETH | 4r | C316H, V321I, E327A | NA | none |

**Supporting information**

**S1 Table. Mutation in the NS5B region of HCV**

*NA; not available as Dasabuvir not licensed for this subtype
